# NFIX Circular RNA Promotes Glioma Progression by Regulating miR-34a-5p via Notch Signaling Pathway

**DOI:** 10.3389/fnmol.2018.00225

**Published:** 2018-07-18

**Authors:** Haiyang Xu, Yu Zhang, Ling Qi, Lijuan Ding, Hong Jiang, Hongquan Yu

**Affiliations:** ^1^Department of Oncological Neurosurgery, The First Hospital of Jilin University, Changchun, China; ^2^Department of Neurosurgery, The First Hospital of Jilin University, Changchun, China; ^3^Department of Pathophysiology, Jilin Medical University, Jilin, China; ^4^Department of Radio-oncology, The First Hospital of Jilin University, Changchun, China; ^5^Department of Ophthalmology, China-Japan Union Hospital of Jilin University, Changchun, China

**Keywords:** glioma, NFIX circular RNA, miR-34a-5p, *NOTCH1*, Notch signaling pathway

## Abstract

**Objective**: The present study aimed to explore the association between NFIX circular RNA (circNFIX) and miR-34a-5p in glioma. Furthermore, this study investigated the influence that circNFIX has on glioma progression through the upregulation of *NOTCH1* via the Notch signaling pathway by sponging miR-34a-5p.

**Methods**: We applied five methods, CIRCexplorer2, circRNA-finder, CIRI, find-circ and MapSplice2, to screen for circRNAs with differential expression between three glioma tissue samples and three paired normal tissue samples. The GSEA software was used to confirm whether significantly different pathways were activated or inactivated in glioma tissues. The binding sites between circNFIX and miR-34a-5p were confirmed by TargetScan. QRT-PCR and western blot were used to measure the relative expression levels of circNFIX, miR-34a-5p and *NOTCH* and identify their correlation in glioma. RNA immunoprecipitation (RIP) validated the binding relationship between circNFIX and miR-34a-5p, while the targeted relationship between *NOTCH1* and miR-34a-5p was verified by a dual luciferase reporter assay. Cell viability and mobility were examined by a CCK-8 assay and wound healing assay, and a flow cytometry assay was employed to analyze cell apoptosis. The nude mouse transplantation tumor experiment verified that si-circNFIX exerted a suppressive effect on glioma progression *in vivo*.

**Results**: Twelve circRNAs were differentially expressed between the tissue types. Of those, circNFIX was the sole circRNA to be overexpressed in glioma among the five methods of finding circRNAs. In addition, the Notch signaling pathway was considerably upregulated in tumor tissues compared with the paired normal brain tissues. It was determined that circNFIX acted as a sponge of miR-34a-5p, a miRNA that targeted *NOTCH1*. Downregulation of circNFIX and upregulation of miR-34a-5p both inhibited cell propagation and migration. Furthermore, a miR-34a-5p inhibitor neutralized the suppressive effect of si-circNFIX on glioma cells. Si-circNFIX and miR-34a-5p mimics promoted cell apoptosis. Moreover, it was demonstrated *in vivo* that si-circNFIX could suppress glioma growth by regulating miR-34a-5p and *NOTCH1*.

**Conclusion**: CircNFIX was markedly upregulated in glioma cells. CircNFIX could regulate *NOTCH1* and the Notch signaling pathway to promote glioma progression by sponging miR-34a-5p via the Notch signaling pathway. This finding provided a deeper insight into the function of circNFIX in human glioma cancer progression.

## Introduction

Glioma, a tumor in the central nervous system, has a poor survival rate and high mortality (Zhang Y. et al., 2017). The latest report suggested that the incidence of glioma has increased from 5.9/100,000 people to 6.61/100,000 people between 1973 and 2016 with the application of improved radiological diagnosis (Lu et al., 2017). Despite multiple treatment options, cancer remains one of the leading causes of death worldwide. It is therefore imperative that further research be completed to determine novel molecular targets for enhanced cancer therapy (Wang R. et al., 2017).

Circular RNA (circRNA), a non-coding RNA with considerable regulatory potency, has received increased attention from RNA researchers in recent years (Li et al., 2015). Recently, it has been found that many circRNAs are derived from protein-coding exons and are widely expressed in cancer cells (Salzman et al., 2013). Most circRNAs originate from exons and are located in the cytoplasm (Venø et al., 2015). CircRNAs are formed by back-splicing covalently joined 3’- and 5’-ends (Zhang X. et al., 2017) and play a role in several cellular functions, including protein binding, RNA transport, and the regulation of translation (Zhang X. et al., 2017). Recently, it has been suggested that circRNAs play a critical role in late-stage gastric cancer (Fang et al., 2017). However, the influence of circRNAs in glioma needs further investigation.

MicroRNAs (miRNAs) are small noncoding RNAs that play critical roles in regulating various cellular functions by transcriptional silencing (Bezerra and Latronico, 2014). For instance, Zhou et al. (2017) proposed that miR-224 could target *SMAD4* to promote colorectal cell propagation. It has been reported that miR-203 downregulates *RGS17* to inhibit cell growth in non-small cell lung cancer (Chi et al., 2017). A growing body of research indicates that miRNAs can play key regulatory roles in glioma, uncovering novel biomarkers for glioma therapy. For example, a study by Xu L. et al. (2017) also indicated that miR-543 acted as a tumor suppressor that could inhibit glioma development *in vitro* and *in vivo*. MiR-34a is an essential member of the miR-34 family (Xu H. et al., 2017). Increasing evidence suggested that miR-34a is important in the research of glioma. Silber et al. (2012) verified that miR-34a significantly affected the growth of proneural glioma cells *in vitro* and *in vivo*. Li et al. (2014) found that upregulation of miR-34a inhibited glioma cell viability and promoted apoptosis. Gao et al. (2013)’s study also provided sufficient evidence for miR-34a as a potentially useful factor for predicting the prognosis of glioma. The present study investigates in greater depth the role of miR-34a-5p in glioma.

Recently, a report demonstrated that a crosstalk exists between the Notch signaling pathways and miRNA in the development and progression of tumors (Wang et al., 2010). The Notch signaling pathway exerts an important function in the progression of various types of cancers by regulating cell propagation, apoptosis, and differentiation (Androutsellis-Theotokis et al., 2006). *NOTCH1* was downregulated in oral cancer cells to help gamma-secretase inhibitors inhibit the spread of cancer cell proliferation (Yao et al., 2007). Thirty percent of all non-small cell lung carcinoma cases have increased activity of the Notch signaling pathway, 10% of which are induced by a mutation in the *NOTCH1* gene (Westhoff et al., 2009). Many reports have shown that the overexpression of *NOTCH1* could inhibit cell apoptosis in many cancers, which suggests its importance (Miele and Osborne, 1999; Jundt et al., 2002). Furthermore, previous reports have revealed that miR-34 could inhibit tumors by regulating the Notch signaling pathway (Subramaniam et al., 2012). MiR-34a could regulate liver regeneration and development in rats via the Notch signaling pathway (Wang X. P. et al., 2017), and miR-34a suppressed breast cancer stem cells by suppressing the Notch signaling pathway (Kang et al., 2015). However, the specific functions of Notch signaling pathways in glioma remain elusive.

In this study, we focused mainly on the mechanism by which the circRNA circNFIX regulated the Notch signaling pathway and, therefore, glioma progression by regulating miR-34a-5p. RNA-Seq data were analyzed through CIRCexplorer2, circRNA-finder, CIRI, find-circ and MapSplice2 to screen differentially expressed circRNAs in glioma tissues. Gene Set Enrichment Analysis (GSEA) was performed with data from the Kyoto Encyclopedia of Genes and Genomes (KEGG) database. By combining *in vivo* and *in vitro* experiments, we confirmed that the Notch signaling pathway was activated in glioma tissues. We further examined the expression levels of circNFIX and miR-34a-5p in glioma tissues and cells and verified their association with glioma progression.

## Materials and Methods

### RNA-Seq Data

Five kinds of blob selector were used in the experiment, including CIRCexplorer2, circRNA-finder, CIRI, find-circ and MapSplice2. RNA-Seq revealed the presence and quantity of RNA in a biological sample at a given moment in time by means of next-generation sequencing (NGS), which could avert many limitations of other transcriptomic approaches. The high-throughput sequencing data for three glioma and paired normal brain tissues from three males were downloaded from the Gene Expression Omnibus (GEO) database^1^, a publicly available database. The series accession number was GSE86202, and the platform was GPL16791. Data quality control (QC) was assessed with FastQC. GENCODEv.19 Gene Transfer Format file was used as a transcript reference (GENCODE annotation). We used RNA-Seq to match sequencing data to the genome and then extracted the undirected comparison of fragments, followed by recombination and comparison with the genome.

### Gene Expression Profiles

The DEseq2 package was used to analyze the differentially expressed circRNAs and mRNAs with the threshold set as |log_2_FD*|* > 1 and adjusted *P* < 0.05 (FD: fold change). Through the Venn intersection analysis, 12 common circRNAs with differential expression including circNFIX were identified in glioma tumor tissues. The 10 most significantly upregulated mRNAs including *NOTCH1* and the 10 most significantly downregulated mRNAs were screened out using the pheatmap package.

### Gene Set Enrichment Analysis

GSEA was performed using data from the KEGG database. The expression data of total normalized mRNAs were uploaded to GSEA v3.0 software. Based on the analysis of GSEA, we also employed the R language “GSEABase” package to perform data processing. We used the ggplot2, DOSE, ggjoy, and clusterProfiler packages to construct the dotplot and joyplot.

### Cell Culture

All cell lines used in the experiment including normal astrocytes HA1800 and human glioma cell lines SF-539, SHG-44 and U87 were obtained from BeNa Culture Collection (Beijing, China). The cell lines were cultured in high-glucose Dulbecco’s Modified Eagle Medium (DMEM) supplemented with 10% fetal bovine serum (FBS) (Invitrogen, Carlsbad, CA, USA), 100 U/ml penicillin and 100 μg/ml streptomycin and then incubated in a humidified atmosphere containing 5% CO_2_ at 37°C.

### Cell Transfection

Si-circNFIX, miR-34a-5p mimics and miR-34a inhibitor were synthesized by GenePharma Co, Ltd. (Shanghai, China). Si-NOTCH1 (#AM16708) was purchased from Invitrogen. The sequence of si-circNFIX is CACACTCCGGGATGAGTTCCA. Glioma cells were placed into a 6-well plate at a concentration of 1 × 10^6^ cells in each well and cultured at 37°C until 90% confluence was reached. Transfections were performed using the Lipofectamine 2000 kit (Invitrogen) according to the manufacturer’s instructions, and the transfection efficiency of the cells was detected after 24 h of incubation.

### RNA Immunoprecipitation (RIP)

A biotin-labeled circNFIX probe (5′-CACCCGTTCATCGAGGCACTGCTG-3′-biotin) was generated by Sangon Biotech Inc. (Shanghai, China). We performed RNA immunoprecipitation (RIP) experiments using the Magna RIP™ RNA-Binding Protein Immunoprecipitation Kit (Millipore, USA) according to the manufacturer’s instructions. U87 cells were first fixed for 10 min using 1% formaldehyde and then lysed and sonicated. Following centrifugation, 50 μl of supernatant was retained and incubated with circNFIX-specific probes-streptavidin Dynabeads (Invitrogen) and blended at room temperature for 24 h. Having washed the Dynabeads-probes-circRNA mixture and incubated it with 200 μl of lysis buffer, we utilized proteinase K to reverse the formaldehyde crosslinking. Lastly, TRlzol was added to the mixture for RNA extraction.

### Luciferase Reporter Assay

The fragment from *NOTCH1* containing the putative binding sites for miR-34a-5p was amplified by PCR, cloned in the firefly luciferase expression vector pMIR-REPORT (Invitrogen) and named *NOTCH1*-WT. To mutate the putative binding sites for miR-34a-5p in *NOTCH1*, the sequence of the putative binding site was replaced as indicated and was named *NOTCH1*-MUT. Before being stably transfected with the pMIR-REPORT-*NOTCH1*-WT and pMIR-REPORT-*NOTCH1*-MUT reporter vectors, together with the Renilla luciferase-expressing vector pRL-TK (Promega, Madison, WI, USA) and the miR-34a-5p mimic or NC using LipofectamineTM 2000 (Thermo Fisher, USA), U87 cells were placed into a 24-well plate at a density of 5 × 10^5^ cells in each well. The relative luciferase reporter activity was detected at 48 h post-transfection.

### QRT-PCR

We used TRIzol (Invitrogen, USA) to isolate the total RNA from cells according to the instructions. Real-time quantitative PCR (qRT-PCR) was used to measure the expression of miR-34a-5p, circNFIX, and *NOTCH1*. We used a TaqMan™ Advanced miRNA cDNA Synthesis Kit (#A28007, Applied Biosystems) to amplify the miRNA. CircRNA and mRNA were amplified with SuperScript™ VILO™ cDNA Synthesis Kit (#11754250, Invitrogen). Total RNA was detected by qRT-PCR using DyNAmo ColorFlash SYBR Green qPCR Kit (#F416XL, Invitrogen). Relative quantification of mRNA expression was normalized by the 2^−ΔΔCt^ method, and GAPDH was used for normalization. All reactions were carried out in triplicate by GeneAmp™ PCR System 9700 (Applied Biosystems). The primers are manifested in Supplementary Table S1.

### Western Blot

Radioimmunoprecipitation assay (RIPA) buffer was utilized to prepare whole-cell lysates. Equal amounts of total protein (30 mg) from cell lysates were loaded on a 6% sodium dodecyl sulfate–polyacrylamide gel for electrophoresis, after which they were transferred to a polyvinylidene difluoride membrane (Millipore). We used an enhanced chemiluminescence western blotting detection system (Bio-Rad) for detection. Primary antibodies used were those against NOTCH1 (#ab52627, 1:1000, Abcam, Hong Kong, China), Jagged1 (#ab7771, 1:500, Abcam), Hes1 (#ab71559, 1:500, Abcam), Hes5 (#ab194111, 1:2000, Abcam), and HEY2 (#ab86010, 1:1000, Abcam). Goat anti-rabbit IgG secondary antibodies (ab7090, 1:2000, Abcam) were employed at 37°C for 1.5 h. The full original images have been uploaded as Supplementary Figure S1.

### Cell Propagation Assay

A Cell Counting Kit-8 assay (CCK-8, Dojin, Japan) was used to investigate U87 cell propagation. Cells were inoculated into 96-well plates with a 100-μl suspension in each well. Cells were cultured in DMEM medium in a 5% CO_2_ incubator for 24 h. The OD values at different time points (24 h, 48 h, 72 h, 96 h and 120 h) were measured at 450 nm using a microplate reader (Varioskan Flash, Thermo Fisher) after adding the CCK-8 solution.

### Apoptosis Analysis

Cell apoptosis was determined by means of a flow cytometer (FACS Calibur, BD, USA) based on the manufacturer’s guidelines. After a 48-h transfection, we used flow cytometry to analyze the apoptosis rate between the control group, NC, si-circNFIX, mir-34a mimics, miR-34a-5p inhibitor and si-circNFIX + miR-34a-5p inhibitor cells, which were then washed and resuspended. We used FACS Diva (BD, USA) to analyze experimental data, and every experiment was done in triplicate.

### Wound Healing Assay

Cells were cultured in 6-well plates for 24 h to form a confluent monolayer. We used a sterile pipette tip (200 ml) to scratch the monolayer, washed the cells twice with PBS and incubated the cells in serum-free DMEM medium. Plates were photographed by a microscope (200×, Olympus) at 0 h and 48 h after scratching at an identical location, respectively, with the width of the scratch measured. The wound area was calculated as width_48 h_/width_0 h_ × 100%. All experiments were performed in triplicate.

### Transwell Migration Analysis

We used 24-well chambers with an 8-μm pore size (Corning, USA) to study cell migration. A total of 5 × 10^4^ cells in 250 μl of DMEM containing 0.2% FBS were placed into the upper chamber, and 500 μl of DMEM (10% FBS) was supplemented into the lower chamber, followed by incubation overnight. Then, cells on the upper side of the well were removed. We fixed the wells in methanol for 20 min and used crystal violet to stain the cells. For quantification analysis, we captured photographs of five random fields. Three identical replicates were performed.

### Tumor Xenograft

We purchased 12 male nude mice (4-week-old) from Shanghai SLAC Experimental Animal Center (Shanghai, China). All the experiments were known and approved by the Ethics Committee of the First Hospital of Jilin University. These male nude mice were assigned to two groups equally (Mock, si-circNFIX). U87 cells transfected with si-circNFIX (2 × 10^6^ cells) were injected into the right limbs of the mice (six mice in each group). After 35 days, all the mice were sacrificed, and then tumors were resected and collected.

### Statistical Analysis

All data analyses were performed using GraphPad Prism 6.0. The above experiments were carried out at least three times. Continuous data were documented as the mean ± standard deviation. The difference between two groups was analyzed by Student’s *t*-test. A *P* value < 0.05 was indicative of statistical significance.

## Results

### CircNFIX Expression and KEGG Pathway Analysis

**Figure 1:**
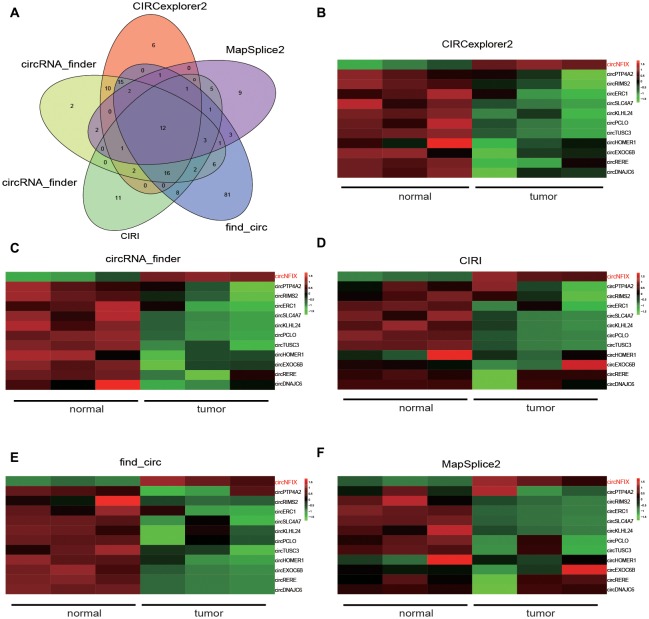
NFIX circular RNA (CircNFIX) expression was overexpressed in glioma tissues compared to paired normal brain tissues. **(A)** Venn intersection analysis selected 12 common differentially expressed circRNA in glioma tissues. **(B)** CIRCexplorer2 analyzed the expression of the 12 genes. **(C)** CircRNA-finder analyzed the expression of the 12 genes. **(D)** CIRI analyzed the expression of the 12 genes. **(E)** Find-circ analyzed the expression of the 12 genes. **(F)** MapSplice2 analyzed the expression of the 12 genes.

**Figure 2:**
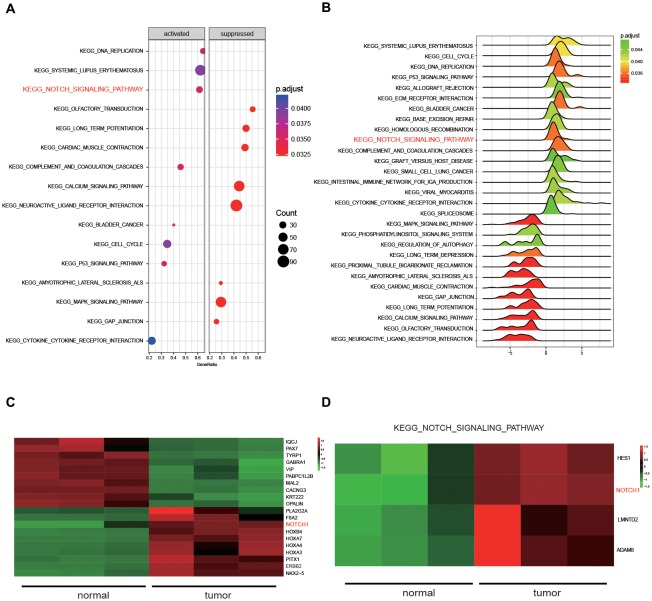
The results of Kyoto Encyclopedia of Genes and Genomes (KEGG) pathway analysis. **(A)** Dotplot analyzed the enrichment of pathways. **(B)** Joyplot analyzed the enrichment of pathways. *P*.adjust value was less than 0.05. **(C)** The heat map presenting the 20 genes that were differentially expressed in glioma tissues and normal tissues. **(D)** The differentially expressed genes in the Notch signaling pathway were selected by intersecting the screened differentially expressed genes from glioma tissues with genes in the Notch signaling pathway.

Five kinds of Blob Selector were used to single out the circRNA that we were interested in, and we found there were 12 common circRNAs that were differentially expressed (Figure 1A). The results of the heat map showed that the 12 circRNAs presented significantly differential expression in tumor tissues, in which circNFIX was confirmed to be overexpressed by all five software packages (Figures 1B–F). The above results suggested that circNFIX was significantly upregulated in glioma tissues. Dotplot displayed 16 significantly different pathways in tumor tissues, of which the Notch signaling pathway was found to be activated in glioma tissues (Figure 2A). Furthermore, Joyplot also further confirmed that the Notch signaling pathway was upregulated in tumor tissues (*P*.adjust < 0.05, Figure 2B). The results of the heat map showed 20 differentially expressed genes (DEGs) including 10 upregulated genes and 10 downregulated genes, of which *NOTCH1* was remarkably overexpressed in glioma tissues in comparison with adjacent normal tissues (Figure 2C). By intersecting the screened DEGs with genes in the Notch signaling pathway, we identified that *NOTCH1* was notably upregulated (Figure 2D). Overall, the Notch signaling pathway was activated in glioma tissues compared with paired normal brain tissues.

### Validation of Relationships Among circNFIX, miR-34a-5p and *NOTCH1*

**Figure 3:**
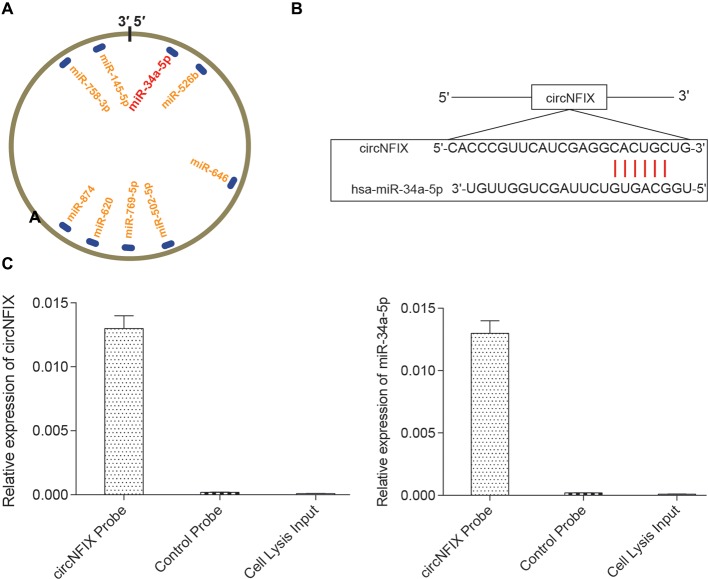
CircNFIX had a predicted binding relationship with miR-34a-5p. **(A)** There were several binding sites on the circRNA. **(B)** CircNFIX had a binding site with hsa-miR-34a-5p. **(C)** The RNA immunoprecipitation (RIP) assay confirmed the targeted relationship between circNFIX and hsa-miR-34a-5p.

**Figure 4:**
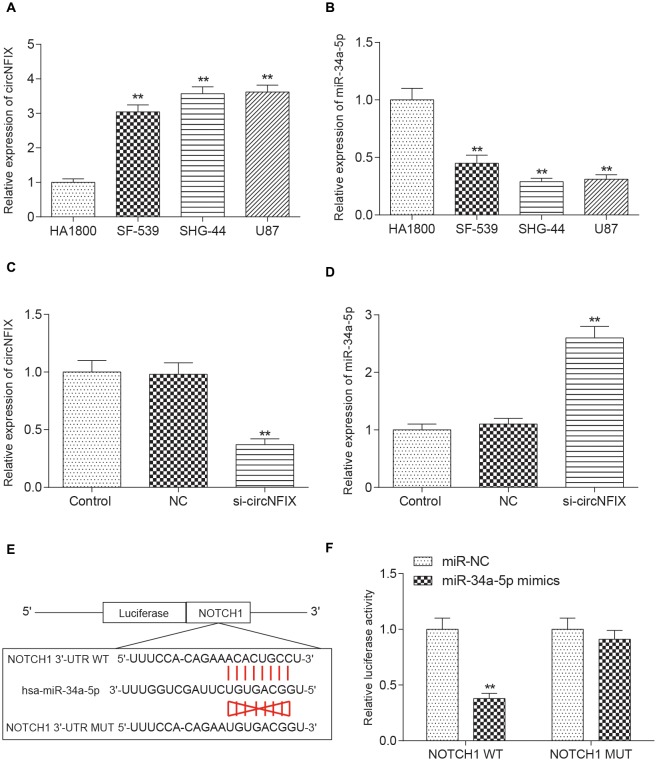
CircNFIX and miR-34a-5p presented differential expression in glioma cells. **(A)** QRT-PCR analyzed the expression of circNFIX in the glioma cell lines SF539, SHG-44 and U87 and the normal cells HA1800. **(B)** QRT-PCR analyzed the expression of miR-34a-5p. **(C)** The expression of circNFIX after transfection with si-circNFIX. **(D)** The expression of miR-34a-5p after transfection with si-circNFIX. **(E)** TargetScan predicted that *NOTCH1* 3’-UTR WT had a binding site for hsa-miR-34a-5p. **(F)** The luciferase reporter assay analyzed the target relationship between *NOTCH1* and miR-34a-5p. **P < 0.01 compared with the NC group.

It is well known that circRNAs function mainly as miRNA sponges to regulate gene expression. We next found the potential miRNAs associated with circNFIX. The result in Figure 3A shows that several miRNAs containing binding sites with circNFIX existed; these miRNAs included miR-34a-5p, miR-526b, miR-646, miR-502-5p, miR-769-5p, miR-620, miR-874, miR-758-3p and miR-145-5p. Of the above miRNAs, miR-34a-5p was the closest to the 5’-UTR, drawing our interest. The binding sites between circNFIX and miR-34a-5p were validated by TargetScan (Figure 3B). Furthermore, in order to verify the relationship between circNFIX and miR-34a-5p in glioma cells, a RIP experiment was performed and confirmed that there was a specific enrichment of circNFIX and miR-34a-5p compared to the controls (Figure 3C). The qRT-PCR results showed that circNFIX was highly expressed in glioma cell lines SF539, SHG-44 and especially U87 cells compared to the normal astrocytes HA1800 (*P* < 0.01, Figure 4A). Meanwhile, the qRT-PCR results indicated that miR-34a-5p was significantly downregulated in glioma cell lines (*P* < 0.01, Figure 4B). Then, we selected the U87 cell line for the following experiments. After transfection with si-circNFIX, the expression of circNFIX was conspicuously decreased, while miR-34a-5p expression was considerably increased compared with the NC group (*P* < 0.01, Figures 4C,D). Additionally, TargetScan revealed that *NOTCH1* 3’-UTR WT had a binding site for hsa-miR-34a-5p, while *NOTCH1* 3’-UTR MUT could not bind to hsa-miR-34a-5p (Figure 4E). Moreover, the luciferase reporter assay also indicated that miR-34a-5p mimics remarkably repressed the relative luciferase activity of *NOTCH1* WT but not *NOTCH1* MUT in comparison with the miR-NC group (*P* < 0.01, Figures 4E–F). Taken together, these findings indicate that circNFIX was upregulated in glioma cells and the expression of miR-34a-5p was significantly increased by knockdown of circNFIX.

### Si-circNFIX and miR-34a-5p Mimics Inhibited the Expression of NOTCH1 and the Downstream Protein Expression in the Notch Signaling Pathway

**Figure 5:**
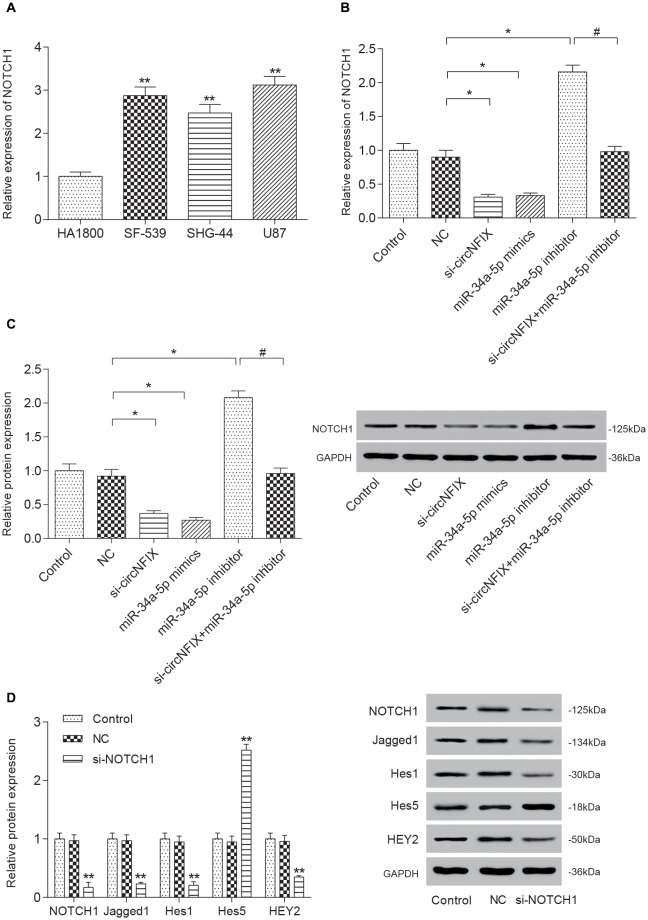
Si-circNFIX and miR-34a-5p mimics inhibited NOTCH1 expression and protein expression downstream of the signaling pathway. **(A)** The qRT-PCR assay analyzed the expression levels of *NOTCH1*. **(B)** The qRT-PCR assay analyzed the expression levels of *NOTCH1* after the U87 cells were transfected with si-circNFIX, miR-34a-5p mimics and the miR-34a-5p inhibitor. **(C)** Western blotting analyzed the protein expression of NOTCH1 after the transfection. **(D)** Western blotting analyzed the protein expression in the signaling pathway. *P < 0.05, **P < 0.01 compared with the NC group, ^#^*P* < 0.05 compared with the si-circNFIX group.

The qRT-PCR results suggested that *NOTCH1* was overexpressed in glioma cells compared with the HA1800 cell line, especially in U87 cells (*P* < 0.01, Figure 5A). Si-circNFIX and miR-34a-5p mimics suppressed the expression of *NOTCH1*, while the miR-34a-5p inhibitor promoted the expression of *NOTCH1*. Si-circNFIX could reverse the facilitative effects of the miR-34a-5p inhibitor on *NOTCH1* expression (*P* < 0.05, Figure 5B). Similarly, the western blot results suggested that miR-34a-5p mimics and si-circNFIX suppressed the expression of the NOTCH1 protein, while the miR-34a-5p inhibitor promoted the expression of the NOTCH1 protein (*P* < 0.05, Figure 5C). The downstream proteins Jagged1, Hes1 and HEY2 in the Notch signaling pathway were significantly downregulated in U87 cells after knocking down circNFIX, while the protein level of Hes5 was increased. It is probable that Notch-Hes signaling may have different impacts, depending on the glioma cell type or differentiation stage of the precursor cell (Wu et al., 2003; *P* < 0.01, Figure 5D). These results suggested that circNFIX could regulate NOTCH1 at both the mRNA and protein levels by acting as a sponge for miR-34a-5p.

### Si-circNFIX and miR-34a-5p Mimics Inhibited Cell Migration, Proliferation and Promoted Cell Apoptosis

**Figure 6:**
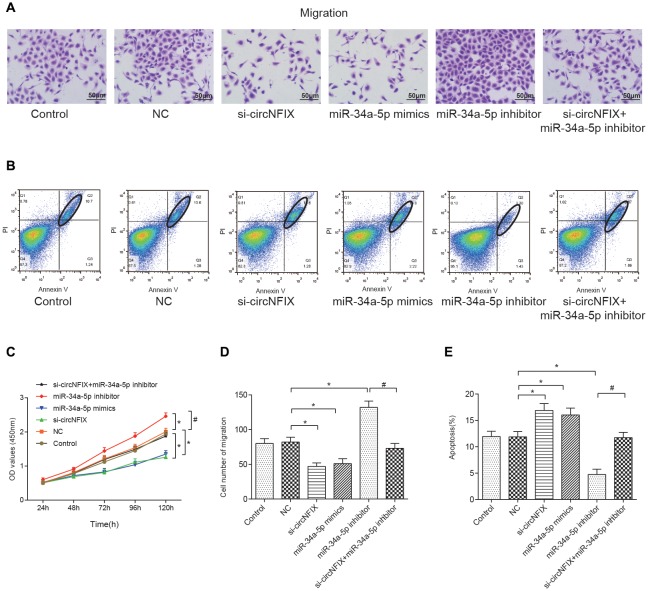
Si-circNFIX and miR-34a-5p mimics inhibited cell migration and proliferation and promoted cell apoptosis. **(A)** The migration assay analyzed cell migration after the transfections. **(B)** Flow cytometry analyzed cell apoptosis after the transfections. Within the ellipse are apoptotic cells. **(C)** The CCK8 assay analyzed cell proliferation. **(D)** The cell migration cartogram presents cell migration levels. **(E)** The cell apoptosis cartogram presents cell apoptosis levels. *P < 0.05 compared with the NC group, ^#^*P* < 0.05 compared with the si-circNFIX group.

The wound healing assay is often used to estimate the coordinated movement of a cell population (Rodriguez et al., 2005). The results of the wound healing suggested that si-circNFIX and miR-34a-5p mimics could inhibit cell migration and propagation, the miR-34a-5p inhibitor could promote wound healing, and the miR-34a-5p inhibitor could alleviate the suppressive effect of si-circNFIX on cell propagation (Supplementary Figure S2). The migration assay showed that si-circNFIX and miR-34a-5p mimics suppressed cell migration compared with the NC condition. The miR-34a-5p inhibitor could alleviate the suppressive impact of si-circNFIX (*P* < 0.05, Figures 6A,D). As the flow cytometry assay showed, si-circNFIX and miR-34a-5p mimics could promote cell apoptosis compared with NC. Similarly, miR-34a-5p could alleviate the suppressive impact of si-circNFIX (*P* < 0.05, Figures 6B,E). The CCK8 assay results suggested that the si-circNFIX and miR-34a-5p mimics suppressed cell proliferation and miR-34a-5p inhibitor promoted cell proliferation (*P* < 0.05, Figure 6C). All above results demonstrate that downregulation of circNFIX and overexpression of miR-34a-5p could suppress cell propagation and promote apoptosis.

### Si-circNFIX Inhibited the Tumor Growth of Glioma *in Vivo*

**Figure 7:**
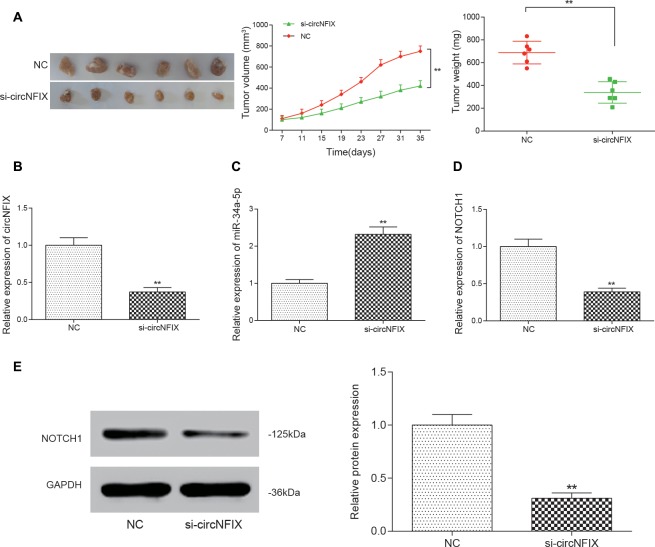
Si-circNFIX inhibited the progression of glioma *in vivo*. **(A)** The nude mouse tumor transplantation experiment proved the suppressive effect of si-circNFIX on the growth of glioma. **(B–D)** The qRT-PCR assay analyzed the expression of circNFIX, miR-34a-5p and *NOTCH1* after U87 cells were transfected with si-circNFIX. **(E)** Western blotting analyzed the protein expression after transfection with si-circNFIX. **P < 0.01 compared with the NC group.

All above experiments demonstrated that si-circNFIX could regulate *NOTCH1* to promote glioma progression by sponging miR-34a-5p *in vitro*. To confirm that knockdown of circNFIX could suppress the tumor growth of glioma *in vivo*, U87 cells transfected with si-circNFIX (2 × 10^6^ cells) were injected into the right limbs of mice. Then, we found that the tumor volume and tumor weight containing si-circNFIX was significantly lower than those containing NC (Figure 7A). Then, the tumor tissues taken out from mice were used to detect the expression of circNFIX, miR-34a-5p and *NOTCH1*. The qRT-PCR results showed that the expression of circNFIX and *NOTCH1* was significantly reduced in the si-circNFIX group compared with NC group, while miR-34a-5p was remarkably upregulated after treatment with si-circNFIX. These results indicated that si-circNFIX could repress the expression of circNFIX and *NOTCH1* but promoted the expression of miR-34a-5p (*P* < 0.01, Figures 7B–D). Western blotting showed that circNFIX suppressed the expression of the NOTCH1 protein relative to that in the NC group (*P* < 0.01, Figure 7E). The results suggested that si-circNFIX exerted an inhibitory influence on glioma progression *in vivo*.

## Discussion

RNA-Seq, as a revolutionary tool for transcriptomics, has been applied in many experiments. For example, Mortazavi et al. (2008) predicted fusion transcripts based on the gene fingerprint profiles of the RNA-Seq paired-end reads (Li et al., 2017). Trapnell et al. (2009) discovered splice junctions with RNA-Seq.

Until recently, circRNAs have largely been considered insignificant by the scientific community due to low expression levels (Chen et al., 2016). However, several research breakthroughs have provided profound evidence that they play major roles in biological functions. For example, evidence suggests that they play a key role in gene regulation, suggesting that they could make viable therapeutic targets in diseases such as cancer (Liu et al., 2016). Therefore, circRNAs have received significantly greater attention in recent years in the study of cancer. For example, Guarnerio et al. (2016) discovered that fusion-circRNAs could both allow cellular transformation and promote cell proliferation and had a tumor-promoting impact in cancer. Zhang H. et al. (2017) found that circRNA UBAP2 (circUBAP2) expression was increased in human osteosarcoma tissues. Li et al. (2018) identified that circ_0046701 was overexpressed in glioma. The study conducted by Yang et al. (2016) also clarified that knockdown of cZNF292 circular RNA could suppress tube formation by inhibiting glioma cell proliferation. Much is still unknown about the exact roles circRNAs may play in glioma development. Thus, we completed an RNA-Seq analysis comparing circRNA expression between glioma and matched adjacent normal tissue to better understand their role in glioma cells. Five methods, including CIRCexplorer2, circRNA-finder, CIRI, find-circ and MapSplice2, were used to screen out differentially expressed circRNAs. Via a Venn diagram, we found circNFIX was upregulated in all five methods. The focus of our project is to determine the role circNFIX plays in glioma progression in addition to uncovering the underlying mechanisms that drive it.

As some of the noncoding RNAs, especially miRNAs, can be biomarkers for glioma diagnosis and treatment (Ahir et al., 2017), we also studied the function of a miRNA (miRNA-34a-5p) that played a role in glioma progression affected by circNFIX. Numerous studies have reported an important role of miR-34a in inhibiting various cancers, and hence, it is often deemed as a tumor suppressor gene (Chen et al., 2015). For instance, Guessous et al. (2010) confirmed that miR-34a was downregulated in glioma tumors. Gao et al. (2013) found that glioma tissues had lower miR-34a expression than normal brain tissues did. In our current study, the expression level of miR-34a-5p was also downregulated in glioma, which suppressed the expression of the genes downstream of the signaling pathway.

A better understanding of the mechanism of circRNAs and miRNAs acting on glioma is meaningful for the discovery of effective therapies against glioma. Specifically, a large number of circRNAs have been verified to sponge miRNAs, thereby functioning as miRNA inhibitors. Bezzi et al. (2017) proposed that circRNA could function as a competing endogenous RNA, in which different RNAs could regulate each other by means of competing for miRNAs through miRNA recognition elements. As Han et al. (2017) reported that the circRNA circMTO1 acted as a sponge of microRNA-9 to repress liver cancer progression. Liang et al. (2017) confirmed the sponge effect of circ-ABCB10 on miR-1271 in breast cancer. Militello et al. (2017) also verified that in addition to lncRNAs, circRNAs could also function as miRNA sponges. These results were consistent with our findings. In our study, circNFIX could suppress glioma progression by sponging miR-34a-5p. However, recent studies had questioned the sponge role that circRNAs play on miRNAs and reported that circRNAs could be translated or take part in tumor progression through a variety of methods. For instance, Legnini et al. (2017) and Pamudurti et al. (2017) reported that circRNAs could function as a eukaryotic endogenous circular RNAs and encode proteins. This may be due to their property that limits the election of the target region for duplication and further restriction of the parameters commonly used for designing mRNA-targeting siRNAs. In this study, we investigated circNFIX expression and its potential role as a microRNA sponge *in vitro* and *in vivo*, further supporting the sponge role of circRNAs.

Furthermore, we discovered that miR-34a-5p suppressed glioma progression *in vitro* by targeting the Notch signaling pathway, which was consistent with results that Jin et al. (2017) reported. In addition, Ji et al. (2008, 2009) found that miR-34 played an important role in cancer stem cell maintenance and survival. The above results all demonstrated that miR-34 could effectively regulate the Notch signaling pathway.

However, this study has several limitations. First, our study was based on U87 cells. Though the U87 cell line is a representative glioma cell type, primary glioma cells derived from patients will be used in our future research. Second, additional mechanisms of circNFIX in regulating glioma progression require further study.

Our research into the oncogenic role of circNFIX in glioma progression has uncovered possible mechanisms through which circRNAs influence cancer growth. However, due to the limited number of samples used in this study, we fully acknowledge that there may be additional circRNAs that play a key role that were not discovered in our data. However, our results suggest that the study of dysregulated circRNAs in cancer can prove fruitful to better understanding how to enhance cancer therapies.

## AUTHOR CONTRIBUTIONS

HX contributed to the study design. YZ and LQ contributed most to experiments practice and data collection. LD and HY helped to analyze data and HJ mainly wrote the manuscript. All authors contributed to the revision and checked the manuscript.

## Conflict of Interest Statement

The authors declare that the research was conducted in the absence of any commercial or financial relationships that could be construed as a potential conflict of interest.

## Footnotes

^1^https://www.ncbi.nlm.nih.gov/geo/

## Supplementary Material

FIGURE S1 Full original images of western blots. The expressions of NOTCH1, HEY2, Hes5, Hes1 and Jagged1 were accessed by western blot.

FIGURE S2 The wound healing assay analyzed cell proliferation after the transfections. **P* < 0.05 compared with the NC group, ^#^*P* < 0.05 compared with the si-circNFIX group.

TABLE S1 Primers for qRT-PCR.
