# Supplementary figures and images for: NFIX Circular RNA Promotes Glioma Progression by Regulating miR-34a-5p via Notch Signaling Pathway
Source: Front Mol Neurosci. 2018 Jul 18;11:225. doi: 10.3389/fnmol.2018.00225 (PMC6058096; doi:10.3389/fnmol.2018.00225)

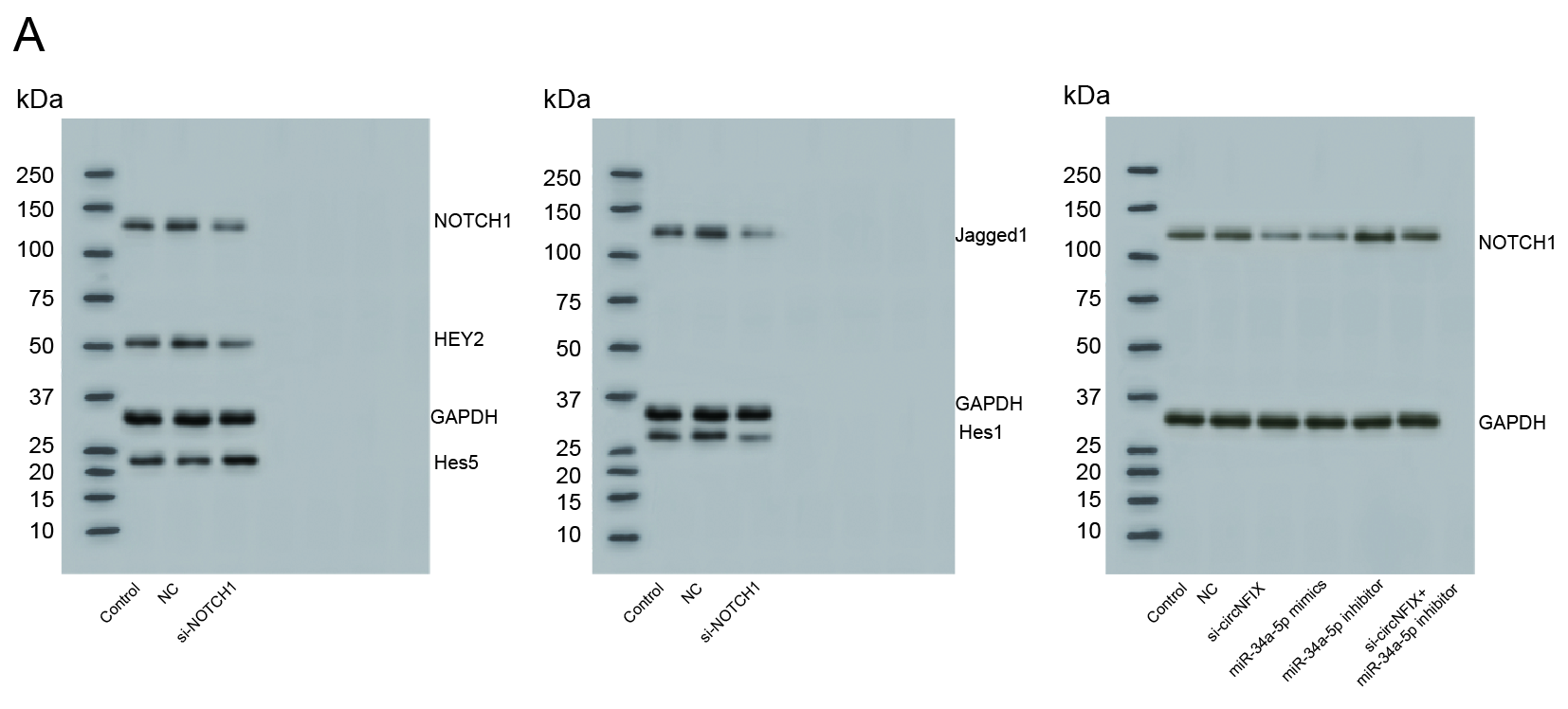

Supplement: FIGURE S1 — Full original images of western blots. The expressions of NOTCH1, HEY2, Hes5, Hes1 and Jagged1 were accessed by western blot. [file Image_1.TIF]

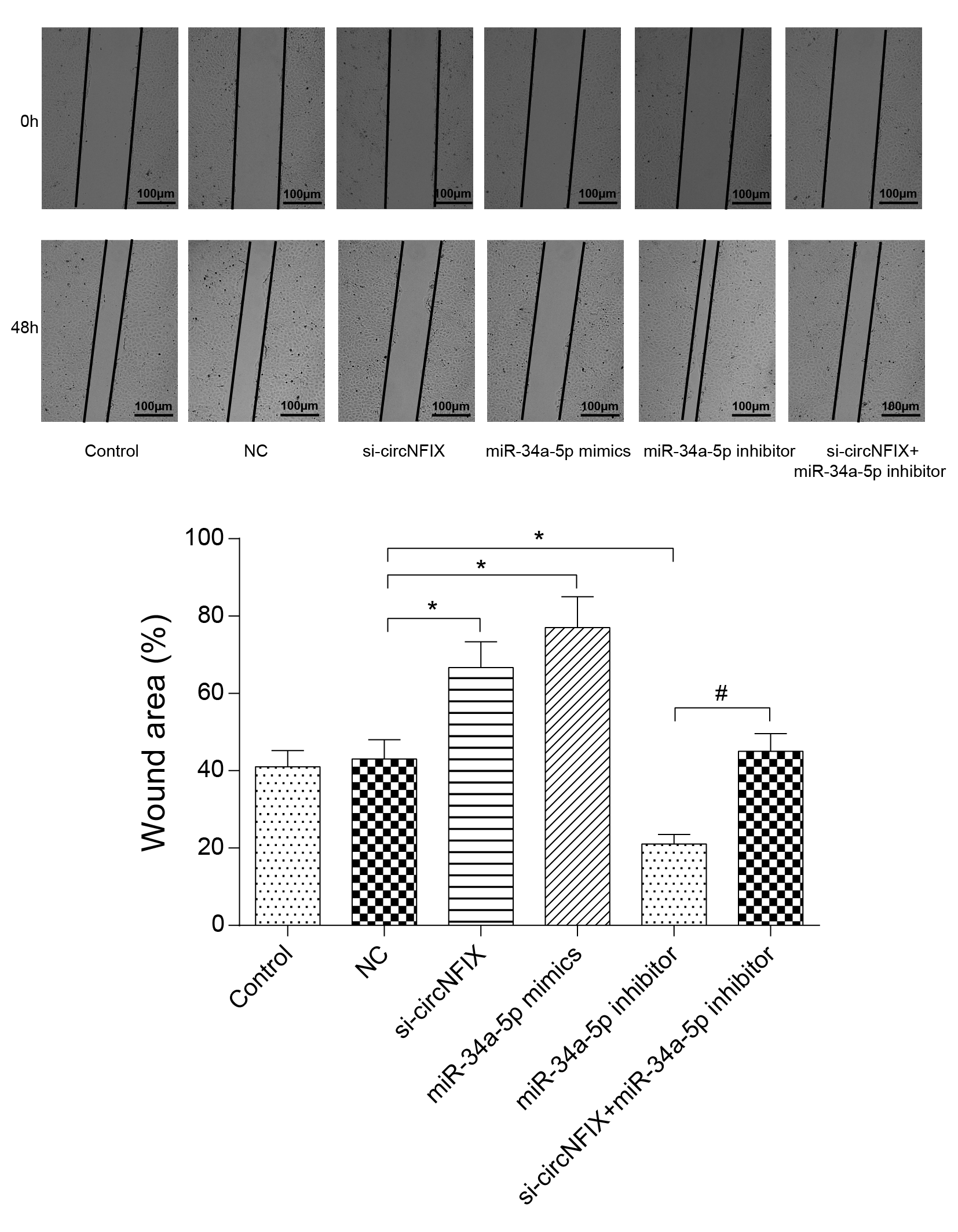

Supplement: FIGURE S2 — The wound healing assay analyzed cell proliferation after the transfections. *P < 0.05 compared with the NC group, #P < 0.05 compared with the si-circNFIX group. [file Image_2.TIF]
