# Supplementary material for: NFIX Circular RNA Promotes Glioma Progression by Regulating miR-34a-5p via Notch Signaling Pathway
Source: Front Mol Neurosci. 2018 Jul 18;11:225. doi: 10.3389/fnmol.2018.00225 (PMC6058096; doi:10.3389/fnmol.2018.00225)
Supplement: TABLE S1 — Primers for qRT-PCR. [file Table_1.DOCX]

**Table 1 Primers for qRT-PCR.**

| **Name** | | **Forward Primers** | | **Reverse Primers** |
| --- | --- | --- | --- | --- |
| **circNFIX miR-34a-5p**  **NOTCH-1**  **GAPDH** | CCTCAGTGCTCGAACCCC TTTGGTCGATTCTGTGACGGT  GCCTTCGTGCTCCTGTTCTT  GGCACAGTCAAGGCTGAGAATG | | CGATGAACGGGTGGAACTCA  Involved in the kit  TCTCTCCGCTTCTTCT ATGGTGGTGAAGACGCCAGTAC | |
